# Implications of Large Language Models for Clinical Practice: Ethical Analysis Through the Principlism Framework

**DOI:** 10.1111/jep.14250

**Published:** 2024-12-01

**Authors:** Richard C. Armitage

**Affiliations:** ^1^ Academic Unit of Population and Lifespan Sciences, School of Medicine, Clinical Sciences Building University of Nottingham Nottingham Nottinghamshire UK

## Abstract

**Introduction:**

The potential applications of large language models (LLMs)—a form of generative artificial intelligence (AI)—in medicine and health care are being increasingly explored by medical practitioners and health care researchers.

**Methods:**

This paper considers the ethical implications of LLMs for medical practitioners in their delivery of clinical care through the ethical framework of principlism.

**Findings:**

It finds that, regarding beneficence, LLMs can improve patient outcomes through supporting administrative tasks that surround patient care, and by directly informing clinical care. Simultaneously, LLMs can cause patient harm through various mechanisms, meaning non‐maleficence would prevent their deployment in the absence of sufficient risk mitigation. Regarding autonomy, medical practitioners must inform patients if their medical care will be influenced by LLMs for their consent to be informed, and alternative care uninfluenced by LLMs must be available for patients who withhold such consent. Finally, regarding justice, LLMs could promote the standardisation of care within individual medical practitioners by mitigating any biases harboured by those practitioners and by protecting against human factors, while also up‐skilling existing medical practitioners in low‐resource settings to reduce global health disparities.

**Discussion:**

Accordingly, this paper finds a strong case for the incorporation of LLMs into clinical practice and, if their risk of patient harm is sufficiently mitigated, this incorporation might be ethically required, at least according to principlism.

The release of ChatGPT on 30 November 2022 [1] elevated large language models (LLMs)—a form of generative artificial intelligence (AI)—to the forefront of public attention. Since then, the potential applications of ChatGPT and other LLMs in medicine and health care have been increasingly explored by medical practitioners and health care researchers. This Research Article considers the ethical implications of LLMs for medical practitioners in their delivery of clinical care through the widely‐influential ethical framework of principlism. A short explanation of LLMs and their potential applications in clinical practice will be followed by a brief discussion of principlism, before an examination of the implications of LLMs on each of the four principles that constitute principlism.

## 1 LLMs

LLMs are a type of deep learning model that are trained on a vast corpus of textual data with which they identify patterns and structures of human language. They have the ability to generate novel human‐like text in response to textual prompts that are inputted by a human user. OpenAI's ChatGPT, a model based on the GPT‐3.5 architecture, was trained on a large data set of conversational text including digital chat logs and social media dialogue with which it learned the nuances of natural language conversation including slang, colloquialisms, and other informal language [1]. While ChatGPT was the first LLM to be made publicly available, a rich landscape of publicly‐available, accessible and user‐friendly LLMs quickly emerged which, as of March 2024, includes OpenAI's GPT‐4 [2], Anthropic's Claude‐3 [3], Google's Gemini [4], Meta's Llama‐2 [5], and xAI's Grok [6].

The ability of LLMs to ‘understand’ and generate human‐like text when applied to text‐based tasks in medicine and health care has and continues to be explored by medical practitioners and health care researchers. GPT‐3.5 has been found to perform to an equivalent standard of a passing third‐year medical student in the US [7], and at or near the passing threshold for all three parts of the US Medical Licensing Exam without any specialised training or reinforcement [8]. It also generated predominantly accurate and comprehensive answers in response to medical questions across 17 specialties as judged by academic physician specialists [9], while the next, and more powerful, iteration of this model (GPT‐4) has been shown to perform strongly when answering postgraduate medical examination questions across various specialties including general practice [10], paediatrics [11], and women's health [12]. With this foundation of medical ‘knowledge,’ the potential utility of LLMs (including ChatGPT) has been recognised in medical education [13], in generating scholarly content [14], in contributing to peer review [15], in parsing unstructured clinical notes [16], in proving antimicrobial prescribing advice [17], and in writing discharge summaries [18], clinic letters [19], and simplified radiology reports [20]. Furthermore, the potential for LLMs to improve health care and health outcomes in low‐ and middle‐income countries has also been outlined [21, 22].

## 2 Principlism

Principlism is the normative ethical framework of professional ethics that was devised by Beauchamp and Childress [23]. This framework, which was designed to aid ethical decision‐making by medical practitioners in health care contexts, comprises of four basic and universal ethical principles that state prima facie moral obligations that are equally important for doctors in the provision of patient care. The ‘Four Principles’ of principlism are the duties of beneficence, non‐maleficence, respect for autonomy, and justice. Since their introduction, the Four Principles have become the primary method for the teaching and evaluation of medical ethical dilemmas in health care contexts and [24], due to their strong support [25, 26, 27, 28], are still widely used today.

An ethical analysis shall now be conducted which examines the implications of LLMs on each of the Four Principles. The analysis shall assume that medical practitioners deploy LLMs as tools to support their delivery of patient care, rather than delegate or entirely hand over clinical decision‐making responsibilities to these technologies. This is due to the inherent limitations of even the most powerful LLMs (including those which might outperform human medical practitioners in the generation of technically correct answers to clinical questions), which have been trained on stylised and uncontaminated vignettes devoid of the complicating factors of real‐world clinical practice, and which do not consider the normative considerations of contextualised individual clinical situations [29]. Therefore, the following analysis shall consider the ethical implications of medical practitioners augmenting themselves with these powerful technologies and wielding them as tools to compound their own clinical reasoning abilities and their delivery of quality patient care. In addition, the analysis shall be restricted to the ethical implications of LLMs being used by medical practitioners in the delivery of patient care, rather than LLMs being used independently by patients in an attempt to improve their own health without the oversight or input of medical practitioners.

## 3 Beneficence

Beneficence requires doctors to act for the benefit of the patient, such as preventing or removing harm, or the active promotion of some good, such as health. In the context of LLMs in clinical practice, if the use of these tools reliably improves patient outcomes, then the duty of beneficence requires that they be incorporated into the delivery of patient care. The ways in which LLMs could be deployed by medical practitioners fall into two broad categories—first, in the support of administrative tasks (such as increasing both the speed and accuracy with which these tasks can be completed) that surround the delivery of patient care, such in the parsing of unstructured clinical notes [16], and in the writing of discharge summaries [18] and clinic letters [19]; second, to directly inform the provision of clinical care, such as in proving antimicrobial prescribing advice [17], and in the answering of knowledge‐based clinical questions across a variety of specialties [9, 10, 11, 12].

The deployment of LLMs by medical practitioners in one or both of these categories might be required to meet the duty of beneficence in at least the following three contexts. First, in low‐resource settings—such as low‐income countries, poorly developed and geographically remote settings, and acute humanitarian contexts—in which the density of highly trained medical practitioners is relatively low. In these locations, the relatively low clinical knowledge base of existing medical practitioners (such as community health workers in isolated rural areas) could be substantially improved by the incorporation of LLMs into their clinical practice. In the absence of alterative means by which the quality of clinical decisions can be increased (such as through discussion with a medical practitioner with superior clinical knowledge, which is often impossible in such settings due to the scarcity of such practitioners), the use of LLMs to up‐skill medical practitioners in their provision of patient care might improve patient outcomes through improved quality, safety and timeliness of care. In addition, the use of LLMs to increase the speed and accuracy with which administrative tasks are completed might also improve patient outcomes by generating more time for the medical practitioner to devote to providing direct patient care, and by improving the efficiency of clinical note communication between different medical practitioners (and within the same medical practitioner over time). Accordingly, the duty of beneficence might require the medical practitioner to augment with LLMs in these settings.

The second context is high‐resource health systems that are under substantial pressure, such as during health emergencies (e.g., during a pandemic), in humanitarian contexts (such as during armed conflict, or in the immediate aftermath of a natural disaster), and during periods of insufficient funding, high staff absenteeism, low staff morale, increasing patient complexity, and growing patient demand (e.g., in the UK's National Health Service [NHS]). In these settings, the density of highly trained medical practitioners is relatively high, although it has been reduced as a result of the particular pressure under which the health system in question is straining. If this has been reduced to such a degree that it induces a negative effect on patient outcomes—such as through elongating waiting times to receive emergency, urgent, and elective medical care (e.g., in the UK's NHS)—LLMs might be used to up‐skill less highly trained medical professionals in a similar fashion to the first context to improve the quality of the care that they deliver, and thereby improve patient outcomes through improved quality, safety, and timeliness of care. In addition, the use of LLMs might also bring about improved patient outcomes by increasing the speed and accuracy with which administrative tasks are completed through the same mechanism as described in the first context. As such, beneficence might require the medical practitioner to augment with LLMs in these settings.

The third context is in all settings, in which even highly trained medical practitioners might improve the quality of their patient care by LLMs directly informing the provision of that care. Even the clinical care provided by medical practitioners who are highly trained might be improved by those practitioners ‘working with’ LLMs, such as by incorporating LLMs into the generation of differential diagnoses [30]. Accordingly, beneficence might require the medical practitioner to augment with LLMs in all settings.

## 4 Non‐Maleficence

Non‐maleficence requires doctors, through their medical interventions, to avoid causing intentional harm to patients. In the context of LLMs in clinical practice, if there is a non‐negligible probability that the use of these tools might cause patient harm and bring about poorer patient outcomes, then the duty of non‐maleficence might require that they not be incorporated into the delivery of patient care. The emerging field of AI ethics aims to identify the key principles that are necessary for the responsible design and application of frontier AI. The World Health Organisation (WHO) 2021 guidance ‘Ethics and governance of AI for health’ translated these principles into the context of medicine and health care, and states that the ethical development and deployment of AI technologies must ‘not result in any mental or physical harm’. [31] With specific regard to LLMs, patient harm might be brought about by these technologies in at least the following four ways.

First, since LLMs produce text that reflects the data sets with which they are trained and the subsequent specialised training and reinforcement that they are subjected to, they have the potential to perpetuate any harmful biases that are contained within those stages of training, such as those pertaining to particular groups based on race, sex, language, and culture [32]. This problem is generally known as algorithmic bias. Furthermore, since these models are largely trained on corpuses of data that originate from well‐funded institutions in high‐income, English‐speaking countries, the resulting under‐representation of alternative cultural perspectives will likely generate LLM outputs that reflect the understandings of health and disease of high‐income countries, which might be less relevant to and applicable in other regions of the world [33]. Second, uncertainties with regard to clinical accountability in the use of LLMs might promote irresponsible deployment of these technologies that lead to patient harm. Without clarity around who is ultimately responsible for the consequences of LLM‐informed decisions that pertain to patient care—is it the medical practitioner who uses the LLM, the health system that approves its use, the technology company that owns it, or the computer engineer who developed it—the individual responsibilities of these actors will remain unclear [34]. Third, many LLMs that are developed for use in medicine and health care will be trained on highly sensitive data, such as the electronic health records of large numbers of patients. In this context, both the ethical principles of consent and confidentiality, and the legal issues of data ownership, copyright and privacy, are highly relevant, and the violation of any of these might bring about substantial patient harm. Fourth, in order for LLMs to be trustworthy, they have to achieve high degrees of transparency (the model must identify the sources of the data from which it formulated its responses to user prompts) and explainability (the model must explain the ‘reasoning’ behind the answers it produces). The tendency for LLMs to ‘hallucinate’ (or ‘confabulate’)—the generation of incorrect or non‐sensical content, including falsified academic citations [34]—means their responses can include inaccurate or unsafe information upon which harmful medical decisions might be based [35]. Simultaneously, current LLMs largely function as ‘black boxes’, and explaining their responses poses serious technical challenges [36]. The resulting problem of trustworthiness might lead to harm through the inappropriate use of LLMs by medical practitioners in clinical decision‐making and in the delivery of patient care, and through reduced trust in medical practitioners and the medical profession more broadly by the patients they serve. If these major potential causes of patient harm cannot be mitigated through sufficient and reliable quality control regulations, non‐maleficence might require the medical practitioner to avoid augmenting themselves with LLMs in their clinical practice.

## 5 Autonomy

Autonomy requires the patient's capacity for self‐determination, and their capacity to make independent decisions in the absence of undue pressure, solicitation or coercion, to be respected. In the context of LLMs in clinical practice, this requires the patient to be informed that LLMs might be used by the medical practitioners that are providing patient care, such that the consent that the patient provides to receive that care can be adequately informed (the WHO 2021 guidance states that ‘the use of machine‐learning algorithms [such as LLMs] in diagnosis, prognosis and treatment plans should be incorporated into the process for informed and valid consent’) [31]. Such transparency is required to both uphold patient autonomy and maintain trust between patients and the medical practitioners (and the medical profession more broadly) that care for them. This requires medical practitioners to explain the manner in which LLMs will be deployed in their care (specifically, that LLMs will be deployed by the medical practitioner as tools in the delivery of patient care, rather than those practitioners delegating or entirely handing over clinical decision‐making responsibilities to these technologies), the reasons of beneficence for doing so, and the potential threats to non‐maleficence that they pose. The patient must understand, retain, weight up, and communicate back to the medical practitioner this information in order for the consent that they provide to receive medical care that is influenced by LLMs to be informed. Respect for autonomy requires that they must be freely able to provide or not provide such consent, and the medical practitioner must be able to provide medical care without augmentation with LLMs if the patient does not consent to the involvement of these technologies. This requirement of respect for autonomy is particularly the case in an ‘adoption phase’ during which LLMs become increasingly incorporated into clinical practice, but might become less relevant if LLMs become firmly established in routine clinical practice and are broadly accepted by patients, medical practitioners, and health systems alike.

## 6 Justice

The principle of justice requires doctors to ensure that the benefits and costs of actions are fairly distributed between patients. One mechanism by which the use of LLMs as tools by medical practitioners in clinical practice might promote justice is through the standardisation of care (the intention to ensure that services provided to patients deliver the desired health outcomes consistent with current clinical knowledge) [37]. This might be achieved in at least two ways: first, the use of LLMs by medical practitioners could mitigate any bias harboured by individual medical practitioners, which might otherwise negatively influence the quality of care provided by the practitioner to members of particular groups. While the problems of algorithmic bias towards particular groups discussed earlier must still be resolved, the use of LLMs as tools in the delivery of patient care could serve to reduce the negative impacts of any bias held by individual medical practitioners by promoting the standardisation of each practitioner's care across all the patients they encounter, and by protecting the quality of that care against the undue influence of any biases held by the individual practitioner. Second, care could be standardised by mitigating the impact of human factors in health care settings that diminish the quality of care delivered by medical practitioners. The potential for human factors, such as the stress and fatigue experienced by medical practitioners under particular conditions (e.g., during night shifts), to negatively impact upon patient safety is well recognised [38]. The deployment of LLMs—which are insusceptible to human factors such as tiredness, hunger, and decision fatigue—could therefore mitigate these factors through their reliably consistent and indefatigably standardised responses to the medical practitioner's prompts, and would therefore promote justice just ensuring a consistent quality of care for all patients at all times (including, e.g., during the medical practitioner's night shifts).

Another mechanism by which the use of LLMs could promote justice is in the context of global health. The density of highly trained medical practitioners is substantially lower in low‐ and middle‐income countries than in high‐income countries [39], and also in geographically remote setting and acute humanitarian contexts. As discussed earlier, the scarcity of highly trained medical practitioners, and the relatively low clinical knowledge base of the existing medical practitioners (such as community health workers in isolated rural areas), in these locations could be substantially enhanced by the incorporation of LLMs into their clinical practice. This is a clearly feasible means by which existing medical practitioners could be up‐skilled due to the wide availability and relative low cost of LLMs (certainly relative to the cost of educating and employing a highly trained medical practitioner) [21, 22]. Through this democratisation of medical ‘expertise’, the negative impact of existing global disparities in the density of highly trained medical practitioners and, consequently, of both the quality of patient care and patient outcome, could be reduced.

Both of these mechanisms by which justice might be promoted are subjected to the requirements of the duty of non‐maleficence. Accordingly, if the risks of patient harm posed by the use of LLMs as tools by medical practitioners cannot be adequately mitigated, their deployment would not be ethically permissible. Indeed, justice might even be violated by the use of LLMs in low resource settings if those LLMs are trained on data generated in high‐income countries and are, therefore, less relevant and applicable, and therefore potentially even harmful, in other regions of the world [33].

## 7 Conclusion

LLMs have recently risen to public prominence, and their potential applications in medicine and health care have and continue to be explored by medical practitioners and health care researchers. This Research Article has considered the ethical implications of LLMs for medical practitioners in their delivery of clinical care through the widely‐influential ethical framework of principlism.

The Article finds that, with regard to beneficence, LLMs can promote health and improve patient outcomes in two ways: first, in the support of administrative tasks that surround the delivery of patient care and, second, by directly informing the provision of clinical care. Accordingly, beneficence might require medical practitioners to incorporate LLMs in their clinical practice in low‐resource settings, in high‐resource health systems that are under substantial pressure, and in all settings in which even highly trained medical practitioners might have the quality of their care improved. With regard to non‐maleficence, LLMs can cause harm through training set bias, uncertainties around clinical accountability, data privacy issues, and insufficient trustworthiness (through poor transparency and explainability). Accordingly, non‐maleficence would prevent the deployment of LLMs if these risks cannot be sufficiently mitigated. With regard to autonomy, medical practitioners must inform patients if their medical care will be influenced by LLMs for the consent that they provide to be informed, and patients must be freely able to choose to provide this consent, while alternative care that is not influenced by LLMs must be available for patients who choose not to provide consent for the incorporation of LLM into the care that they receive. Finally, with regard to justice, the use of LLMs by medical practitioners could promote the standardisation of care, both through the mitigation of any biases harboured by the individual medical professional, and through the LLM's invulnerability to deleterious human factors such as stress and fatigue. In addition, the incorporation of LLMs into the clinical practice of lesser skilled existing medical practitioners in low resource settings could reduce global disparities in quality of care and patient outcomes.

## 8 Recommendation for Practitioners

As such, this Article finds that there is a strong case for the use of LLMs as tools by medical practitioners to augment their clinical practice. There are, however, substantial risks of patient harm posed by the deployment of LLMs in this manner. Should these risks be sufficiently mitigated, such as through sufficient and reliable quality control regulations, their incorporation into clinical practice might be ethically required, at least according to the ethical framework of principlism. However, further analysis that considers a broader range of ethical principles and concepts beyond the constituents of principlism is required before any policy recommendations on this matter can be offered.

## 9 Summary of Findings

**Table:** 

Beneficence	Non‐maleficence	Respect for autonomy	Justice
LLMs support administrative tasks that surround patient care. LLMs directly inform clinical care.	Non‐representative training data sets. Uncertainty around clinical accountability. Issues around consent, confidentiality, data ownership, and privacy. Patient and clinician trust in LLMs.	Informed consent required if care will be influenced by LLMs. Alternative care uninfluenced by LLMs must be available.	LLMs mitigate clinician bias and protect against human factors, standardising care. LLMs up‐skill clinicians in low‐resource settings, reducing global health disparities.

## Conflicts of Interest

The author declares no conflicts of interest.

## Data Availability

The author has nothing to report.
